# Efficacy and Safety of Electroacupuncture on Treating Depression Related Sleep Disorders: Study Protocol of a Randomized Controlled Trial

**DOI:** 10.1155/2016/1069597

**Published:** 2016-12-29

**Authors:** Xuan Yin, Jian Xu, Bo Dong, Jie Ma, Zeqin Chen, Ping Yin, Junyi Wu, Bochang Zhu, Yan Cao, Huimin Zheng, Lixing Lao, Shifen Xu

**Affiliations:** ^1^Shanghai Municipal Hospital of Traditional Chinese Medicine Shanghai, Shanghai University of TCM, Shanghai 200071, China; ^2^School of Chinese Medicine, The University of Hong Kong, 10 Sassoon Road, Pokfulam, Hong Kong; ^3^Center for Integrative Medicine, School of Medicine, University of Maryland, Baltimore, MD 21201, USA

## Abstract

*Background*. Depression is frequently accompanied by sleep disturbances including insomnia. Insomnia may persist even after mood symptoms have been adequately treated. Acupuncture is considered to be beneficial to adjust the state of body and mind and restore the normal sleep-awake cycle. This trial is aimed at evaluating the efficacy and safety of electroacupuncture on treating insomnia in patients with depression.* Methods*. We describe a protocol for a randomized, single-blinded, sham controlled trial. Ninety eligible patients will be randomly assigned to one of 3 treatment groups: treatment group (acupuncture), control A group (superficial acupuncture at sham points), and control B group (sham acupuncture). All treatment will be given 3 times per week for 8 weeks. The primary outcome is the Pittsburgh Sleep Quality Index (PSQI). The secondary outcomes are sleep parameters recorded in the Actigraphy, Hamilton Rating Scale for Depression (HAMD), and Self-Rating Depression Scale (SDS). All adverse effects will be accessed by the Treatment Emergent Symptom Scale (TESS). Outcomes will be evaluated at baseline, 4 weeks after treatment, 8 weeks after treatment, and 4 weeks of follow-up.* Ethics*. This trial has been approved by the Ethics Committee of Shanghai Municipal Hospital of Traditional Chinese Medicine (2015SHL-KY-21) and is registered with ChiCTR-IIR-16008058.

## 1. Background

Depressive disorders are among the most common psychiatric disorders in adults. Previous study identified major depressive disorder (MDD) as the second leading cause of disability worldwide and results in an enormous public health burden [1]. The estimation of lifetime prevalence of MDD in China is 3.3% [2], causing an urgent need to improve health services for these patients.

Except depressive disorders including negative emotions, anxiety, agitation, low self-esteem, and suicidal tendencies [3], MDD is frequently accompanied by sleep disturbances such as insomnia or hypersomnia [4]. Insomnia can be found in 80–90% of patients with MDD [5], characterized by persistent dissatisfaction with sleep quantity or quality, with specific complaints of difficulty falling asleep, frequent nighttime awakenings, and/or awakening earlier in the morning than desired [6]. For many patients, insomnia persists even after mood symptoms have been adequately treated.

At present, the treatment of comorbid MDD and insomnia is given priority to pharmacological treatment, complemented with cognitive behavioral therapy (CBT), sleep hygiene education, and other treatments [7–9]. Although the development of various classes of antidepressants and sedative-hypnotics, represented by selective serotonin reuptake inhibitors (SSRIs) and barbiturates, has considerably improved the efficacy and prognosis in the treatment, the currently available pharmacotherapy is unsatisfactory [10]. Long-term use of these medications can cause side effects such as excessive sedation, tolerance, addiction, neurological toxicity, or other psychiatric disorders [11, 12].

Acupuncture, as a widely recognized alternative therapy in clinical practice, has been used to treat depressive disorders and related sleep disturbances recently. According to the theory of traditional Chinese medicine, acupuncture provides overall coordination, helping to achieve the state of relative equilibrium of body and mind and to restore the normal sleep-awake cycle. Researchers believe that electroacupuncture (EA) treatment can promote the sleep quality by direct manipulation of autonomic nervous system (ANS) [13]. In addition, some trials have investigated that EA can improve depressive disorders caused by cancer [14] or poststroke [15] and can act on depression by protecting nerve cells in the hippocampus [16]. Although the number of studies on depression or insomnia by acupuncture increases every year, there is little focus on the effects of EA on comorbid depression and insomnia and the quality of these related RCTs remains stagnant due to the methodological limitations that reduce the credibility of the results [17].

To study the clinical effects of electroacupuncture on treating insomnia and alleviating depression and to resolve some logic problems of present acupuncture researches, we design this randomized, parallel-group, and single-blinded clinical trial with a sufficient follow-up period. As a preliminary experiment, we aim at observing the effects of EA treatment on sleep status by subjective and objective assessments and eliminating the placebo effect of EA by setting reasonable sham acupuncture method as well. The results will be helpful to demonstrate if EA is an effective and safe therapy for improving sleep quality and mental health for patients with depression. The findings will be shared with the healthcare professionals, general public, and relevant organizations through publication of manuscripts and conference presentations.

## 2. Methods/Design

### 2.1. Setting and Design

We will conduct a single-center, patient-assessor-blinded, randomized controlled trial to evaluate the efficacy and safety of electroacupuncture for insomnia in patients with depression. The trial will be performed at the acupuncture department in Shanghai Municipal Hospital of Traditional Chinese Medicine in Shanghai, China. We will recruit 90 patients who meet inclusion criteria and randomly assign them to one of 3 groups, receiving electroacupuncture, superficial acupuncture at sham acupoints, or sham acupuncture. After one week of washout period, patients will accept 12 weeks of observation (Table 1). All treatment will be given 3 times a week (every other day) for 8 weeks. Participants will be assessed at 4 time points, the baseline (1 week before treatment), the middle of the treatment (4 weeks after treatment starts), end of the treatment (8 weeks after treatment starts), and follow-up (4 weeks after treatment finishes). All participants will complete the assessments by the PSQI, Actigraphy, HAMD, SDS, and TESS. We will follow the Standards for Reporting Interventions in Clinical Trials of Acupuncture (STRICTA) [18] throughout the trial.

**Figure 1:**
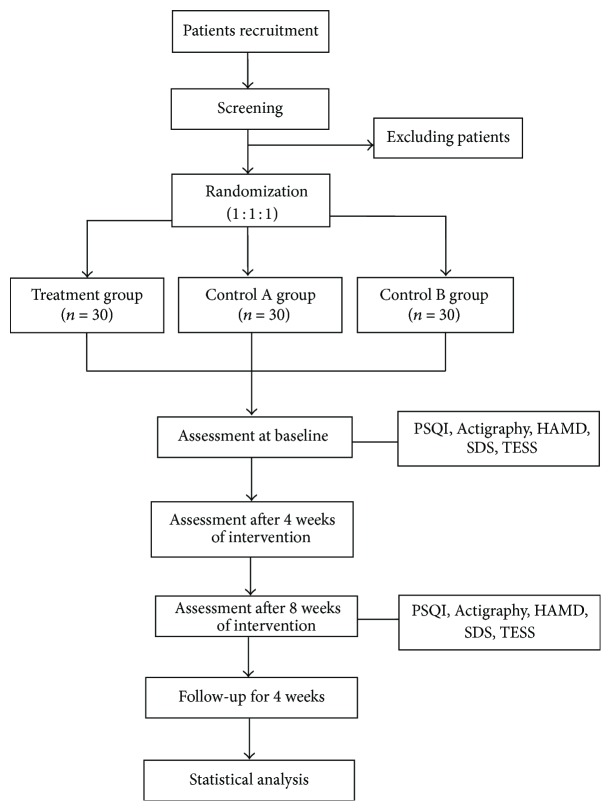
Flowchart of the trial. PSQI: Pittsburgh Sleep Quality Index; HAMD: Hamilton Rating Scale for Depression; HAMA: Hamilton Rating Scale for Anxiety; SDS: Self-Rating Depression Scale; TESS: Treatment Emergent Symptom Scale.

Figure 1 is a flowchart of the study.

#### 2.1.1. Inclusion Criteria

Participants meeting the following criteria will be included:

1. Male or female participants aged 18–70
2. Participants who meet the diagnostic criteria of depression according to the Diagnostic and Statistical Manual of Mental Disorders, Fourth Edition (DSM-IV) [19]
3. Participants whose HAMD score is 20–35
4. Participants who have complaint about insomnia at the first visit to the doctor
5. Participants whose PSQI score is more than 7
6. Participants who voluntarily agree with the investigation and sign a written informed consent form for the clinical trial

#### 2.1.2. Exclusion Criteria

Participants who report any of the following conditions will be excluded:

1. Participants with secondary depressive disorders caused by organic diseases, medicine, or psychotic disorders including schizophrenia
2. Participants who are in the depressive episode of bipolar disorder or suffering from dysthymia, reactive depression, and depressive syndrome caused by other diseases
3. Participants with alcohol abuse or drug dependence
4. Participants who refuse to wear the Actigraphy during the trial
5. Pregnant or lactating women

### 2.2. Sample Size Calculation

As a preliminary trial, the calculation of sample size is based on the open study about acupuncture for aging patients with depression and sleep disorders [20]. Referring to the study, the designer recruited 24 patients in each group to take acupuncture or sham treatment and also used PSQI as the main assessment indicator and suggested further study in controlled trials on the effects of acupuncture for younger patients with depression or insomnia. To study about the efficacy of electroacupuncture on depressive patients with insomnia, we positively take the previous results and 20% dropout rate into consideration and determine that the sample size of each group is 30 participants. With 1 : 1 : 1 allocation to each group, a total of 90 participants should be recruited.

### 2.3. Recruitment

The participants will be recruited through hospital-based advertisements from outpatient clinic and from website of Shanghai Municipal Hospital of TCM. If they have interest in participating, they can take the phone screening first and will be asked for screening visit in the hospital where they are asked to fill in some scales and then wear a wrist Actigraphy to monitor their sleep quality. The Actigraphy will be given to the participants for one evening and returned the next day. The data analyst will collect data from the Actigraphy and evaluate their sleep quality. Once the participants meet the inclusion criteria, they will be asked to sign the written informed consent form before intervention begins.

### 2.4. Randomization and Blinding

We plan to use the simple randomization method in this trial. An independent researcher will use the software SPSS20.0 to generate a random number table with the ratio of 1 : 1 : 1 to divide 90 participants into three groups. Then the researcher will make random allocation cards and seal each card in an opaque envelope whose number is the same as the time sequence in which eligible patients register for the trial. Another researcher will arrange the patients into different groups and tell the acupuncturists about the group assignment.

We will conduct a patient-assessor-blinded trial where participants are not aware of their group assignments and acupuncturists will not be involved in the outcome assessments or data analyses. We will make efforts to validate the successful implementation of the blinding method. Since the participants who have had previous acupuncture can more easily differentiate between real and sham treatment compared to inexperienced individuals [21], our recruiter will ask whether the patients have been treated by acupuncture for depression related insomnia before. Furthermore, participants will be asked to wear an eye mask before and during the treatment. Each treatment will be conducted in a closed unit with drape. Except the acupuncturists, other researchers including the statisticians, outcome assessors, and data analysts are all blinded to the group assignments. All researchers will accept training about the specification of this research method before the trial and strictly adhere to the task separation principle.

### 2.5. Intervention

Participants in three groups will receive different acupuncture or sham acupuncture methods. Participants in each group will receive treatment 3 times a week for 8 weeks. All treatments will be performed after skin cleansing, with patients wearing an eye mask and lying supine. All acupuncturists are licensed doctors with more than 3 years of experience in acupuncture treatment. Each treatment will last for 30 minutes. The temperature of the treatment room cannot be lower than 25°C.

Considering the participants' psychological state, they will be allowed to take regular oral administration of SSRIs. They must record the amount of SSRIs especially when they reduce the amount.

#### 2.5.1. Treatment Group

Participants in the treatment group will receive electroacupuncture treatment. Acupuncture method of each acupoint is shown in Table 2. Regular acupuncture method will be applied at Baihui (GV20), Shenting (GV24), Yintang (GV29), bilateral Anmian (EX-HN22), Shenmen (HT7), Sanyinjiao (SP6), and Neiguan (PC6). After needle insertion, rotating manipulation or lifting-thrusting manipulation will be applied for “Deqi” sensation. The electroacupuncture apparatus (CMNS6-1, Wuxi Jiajian Medical Device Co., Ltd., China) will be connected to the needles at Baihui (GV20) and Yintang (GV29) for 30 minutes and deliver a continuous wave to the patients. The frequency will be set at about 30 Hz and the amplitude will be less than 20 V during the treatment, based on the endurance of each patient.

#### 2.5.2. Control A Group

Participants in control A group will receive superficial acupuncture treatment at the following sham acupoints: bilateral acupoints 0.5 cun upper outer of Jiache (ST6), Waiguan (SJ5), Yangchi (SJ4), and Xuanzhong (GB39); acupoint upper outer of Quanliao (SI18); and bilateral acupoints in the middle of Tianyou (SJ16) and Tianrong (SI17). Acupuncturists will do no manipulation at each acupoint after inserting the needles. The electroacupuncture apparatus will be connected to the needles at bilateral acupoints 0.5 cun upper outer of ST6, with zero frequency and amplitude.

#### 2.5.3. Control B Group

Participants in control B group will receive sham acupuncture treatment at the same acupoints as control A group. The sham acupuncture will be applied with the placebo needles used in some trials before [22, 23]. When the tip of the blunt needles touches the skin, the patient will get a pricking sensation but there is no real needle inserted into the skin. The electroacupuncture apparatus will be set beside the patients, with no connection to the needles. Inform the patients when removing the needles after 30 minutes. Use the dry cotton ball to press the acupoints so that patients can feel the withdrawal of the “real” needles.

### 2.6. Outcome Measurement

#### 2.6.1. Primary Outcome

The Pittsburgh Sleep Quality Index (PSQI) is a widely used questionnaire to assess one's sleep disorders over one month. It is comprised of 19 self-rated items and 5 other-rated items [24]. The scores include the following indicators: subjective sleep quality, sleep latency, sleep duration, habitual sleep efficiency, sleep disturbances, use of medication, and daytime dysfunction. Each indicator is rated from 0 to 3. The accumulated scores of the seven indicators constitute the total score of PSQI (0–21). The higher score indicates the worse sleep quality and severer sleep disorders.

The Secondary outcomes are listed as follows:

#### 2.6.2. The Secondary Outcomes

1. Actigraphy (wActiSleep-BT, Actigraph LLC, Pensacola, USA), which is worn on the patient's wrist, can monitor the quality of sleep, such as sleep onset, sleep latency, total sleep time, sleep awakenings during the night, duration of sleep, and sleep efficiency. The software ActiLife6 (Version 6.8.1, Actigraph LLC) will be used to analyze every participant's sleep condition recorded in the Actigraphy.
2. The Hamilton Rating Scale for Depression (HAMD), an observer-rating questionnaire with 17 items is used to describe the severity of cognitive and bodily symptoms of depressive disorders [25]. Each item is rated in 3- or 5-point scales. The higher total score indicates the severer depression.
3. Self-Rating Depression Scale (SDS) is a self-rated scale to assess the severity of depressive adults [26]. The standard score is the integer part of 1.25 times of the raw score added up by the total 20 questions. The standard score of more than 53 points means the subject has depressive symptoms. The higher the score is, the more serious the depression is.

#### 2.6.3. Adverse Events

Any adverse event (described as unfavourable or unintended signs, symptoms, or diseases occurring during the trial) related to the intervention or administration of SSRIs will be reported by patients and practitioners and accessed by the Treatment Emergent Symptom Scale (TESS) which is used as an associated indicator to mainly evaluate the safety of acupuncture treatment in this trial.

### 2.7. Quality Control

In order to guarantee the quality of the trial, it will be carried out under the supervision of two departments, Shanghai Municipal Hospital of Traditional Chinese Medicine and Shanghai Academy of Education Science. We will promptly input data on the ResMan website. The Clinical Research Center of Drugs of Shanghai University of TCM will work as the data monitoring team to identify problems in the project, examine collected data, and control bias. Meanwhile, a qualified clinical trial expert will be invited to monitor this study.

### 2.8. Statistical Analysis

All analyses will be performed on the Intention-to-Treat (ITT) population of participants who have at least one treatment. Data analyses will be performed with the use of the statistical software SPSS20.0. *t*-test is used to compare the measurement data between the two groups from the baseline to 4 weeks of follow-up; and rank-sum test is used for ranked data, while *χ*^2^ test is adopted to analyze categorical data. The significance level used for statistical analysis with 2-tailed testing was 5%. Data values are mainly presented as Mean ± SD.

### 2.9. Ethics

The trial has been approved by the Ethics Committee of Shanghai Municipal Hospital of Traditional Chinese Medicine, Shanghai, China (2015SHL-KY-21) and is registered with Chinese Clinical Trials Registry (ChiCTR-IIR-16008058). All patients will sign the written informed consent before participating in the trial.

## 3. Discussion

Insomnia has been identified as the most common condition comorbid to depressive disorders [27]. The relationship between insomnia and mood symptoms is bidirectional in that poor sleep can precede an episode of major depressive disorder, and depressive mood can disrupt normal sleep patterns [4]. As research continues, some experts have suggested that comorbid insomnia and depression may have a different clinical course than either condition alone and may thus require specific treatment procedures. Electroacupuncture has been used as a complementary treatment to relieve unhealthy mood [28] and to normalize sleep disturbance [29] but the few studies focus on its efficacy and safety for treating comorbid depression and insomnia.

The main goal of our trial is to present a well-designed trial to study the therapeutic effect of EA on insomnia in depressive patients and overcome some methodological challenges in previous acupuncture clinical researches. Previous RCTs about acupuncture for depression or insomnia had limitations including imperfect blinding method, illogical design, or practical difficulty. To reach the goal, we design this strictly randomized controlled trial, setting two control groups to, respectively, apply sham acupoints and sham acupuncture method so that the result may help other researchers have better understanding about the effects of EA and the certain specificity of acupoints on treating insomnia and alleviating depression. In addition, we use single-blinded method to reduce test bias and ensure the reliability of the research.

Optimizing point selection of acupuncture treatment for insomnia is an important part in this trial. The TCM theory about the specificity of acupoints is one of the most mysterious questions mentioned by the worldwide acupuncture researchers [30]. To give some evidence for the effects of “specific” acupoints in treating depression related insomnia, we set two control groups with acupuncture at “nonspecific” acupoints in this trial, hoping to figure out differences between the two ways of acupoints selection. Combining acupuncture theory with acupuncturists' experience, we regard acupuncture method of regulating the Governor Vessel as the main treatment principle and choose two acupoints (GV20 and GV29) in GV for electroacupuncture to enhance the curative effect for treating insomnia. Imageology study also found that electroacupuncture stimulation at GV20 can modulate the default mode network in patients with depressive disorder, which supports that EA has positive effect on treating comorbid depression and insomnia [31]. The application of the Actigraphy is another flashpoint in the trial. Studies reported that the wrist-worn sleep monitor provides more objective data of sleep quality and duration than patients' self-reported data [32]. It is helpful to compare, analyze, judge, and evaluate changes in sleep quality before, during, and after the treatment. We ask the participants to wear the Actigraphy at night each time we give it to them and to return it next day so that we can ensure the latest data and analyze them in time.

The key technical issues that need to be solved in this trial are (1) the application of the sham acupuncture method and (2) the challenge of patient compliance. All acupuncturists should accept training for several times before the trial begins to ensure the implementation of the sham acupuncture techniques and prepare for reasonable explanation in case of being questioned by patients in control B group. Patients' compliance is a common issue existing in various clinical trials. Good compliance is the central premise to help the trial move forward. Therefore, we will strengthen the publicity and education of medical knowledge to patients and improve healthcare in all aspects. For instance, appointments should be made by phone or email to establish the reservation system; reasonable arrangement of consultation time should be made to increase the patients' attendance rate; professional researchers will conduct family follow-up observation to supervise its implementation; and free acupuncture treatment will be promised by acupuncturists if the patients are not satisfied with the results of their treatment after the trial.

The limitation of this trial is the single-blinded method. Different from the placebo medicine trial, it is inevitable for the acupuncturists to know well about the real or sham acupuncture treatment and its possible effects. This is the reason why we apply the eye mask for the patients, use drape for separation during treatment, and set up training program for the acupuncturists.

We will standardize point selection, manipulation, needling instrument, and therapists' clinical experience in accordance with the STRICTA to ensure quality control of our acupuncture treatment. The objectively recognized assessment will improve the reliability of efficacy evaluation. We expect that this study will provide more credible evidence for the effects of EA on insomnia and depression and make up for the deficiencies in the current methodology of acupuncture clinical trials.

## Figures and Tables

**Table 1:** Trial process chart.

	Baseline	Treatment phase			Follow-up phase
	Week 1	Week 0	Week 4	Week 8	Week 4
*Patients*					
Enrollment	×				
Signed informed consent		×			
Medical history	×				
Merger disease	×				
Randomization		×			
Intervention			×	×	
*Primary outcomes*					
PSQI	×		×	×	×
*Secondary outcomes*					
Actigraphy	×		×	×	×
HAMD	×		×	×	×
SDS	×		×	×	×
TESS			×	×	×
SSRIs dose record	×		×	×	×
Patients' compliance			×	×	×

**Table 2:** Acupuncture method for each acupoint.

Number	Acupoint	Needling method	Needles
1	Baihui (GV20)	The angle between the needle tip and the scalp is 30 degrees. Move the needle tip backward along the anterior-posterior midline, and then insert the needle for 0.5 cun.	0.3 *∗* 40 mm
2	Shenting (GV24)	The angle between the needle tip and the scalp is 30 degrees. Move the needle tip backward along the anterior-posterior midline, and then insert the needle for 0.5 cun.	0.3 *∗* 25 mm
3	Yintang (GV29)	Pinch the local skin, and then puncture obliquely for 0.5 cun	0.3 *∗* 25 mm
4	Anmian (EX-HN22)	The angle between the needle tip and the scalp is 30 degrees. Puncture perpendicularly for 0.5 cun with the needle tip to the nose tip.	0.3 *∗* 40 mm
5	Shenmen (HT7)	Puncture perpendicularly for 0.5 cun.	0.3 *∗* 25 mm
6	Sanyinjiao (SP6)	Puncture perpendicularly for 1 cun.	0.3 *∗* 40 mm
7	Neiguan (PC6)	Puncture perpendicularly for 0.5 cun.	0.3 *∗* 40 mm

## Abbreviations

MDD: Major depressive disorder
STRICTA: Standards for Reporting Interventions in Clinical Trials of Acupuncture
PSQI: Pittsburgh Sleep Quality Index
HAMD: Hamilton Rating Scale for Depression
HAMA: Hamilton Rating Scale for Anxiety
SDS: Self-Rating Depression Scale
TESS: Treatment Emergent Symptom Scale
ITT: Intention-to-Treat Set
GV: Governor vessel
EX-HN: Extra acupoints on head
SI: Small intestine meridian of hand Taiyang
SJ: Sanjiao meridian of hand Shaoyang
SP: Spleen meridian of foot Taiyin
HT: Heart meridian of hand Shaoyin
PC: Pericardium meridian of hand Jueyin
CRF: Case report form.

## Additional Points

This clinical trial is currently recruiting participants.
